# Supplementary material for: Practices of herbal management of malaria among trading mothers in Shai Osudoku District, Accra
Source: PLoS One. 2022 Jul 18;17(7):e0271669. doi: 10.1371/journal.pone.0271669 (PMC9292099; doi:10.1371/journal.pone.0271669)
Supplement: Attachment — Submitted filename: RESPONSE TO EDITOR.docx [file pone.0271669.s001.docx]

RESPONSE TO EDITOR

| COMMENT | RESPONSE |
| --- | --- |
| Please clarify why you obtained approval from The Dodowa Health Research Centre Institutional Review Board (DHRC- IRB) which it is not the affiliation on the Authors | Valley View University which the authors are affiliated to does not have ethical review board, However the IRB which granted permission for the study to be done is under Ghana Health service and is located in the District where the study was conducted |
