# Supplementary material for: Practices of herbal management of malaria among trading mothers in Shai Osudoku District, Accra
Source: PLoS One. 2022 Jul 18;17(7):e0271669. doi: 10.1371/journal.pone.0271669 (PMC9292099; doi:10.1371/journal.pone.0271669)
Supplement: Attachment — Submitted filename: review comments.docx [file pone.0271669.s002.docx]

Plus one journal

| Line/ page/ section | Remarks |
| --- | --- |
| Title | Remove full stop after the title. |
| 4^th^ line of abstract | Change “the researchers, therefore aim … “ to “ The study aims at …” |
| Conclusion in abstract | The conclusion in the abstract is misplaced. Authors must summarize their findings. |
| Lines 3-5 of introduction | Complete the statistics on the number of deaths attributed to malaria. Is it per annum or monthly? |
| Last paragraph of introductory page | Scientific names of trees should be written in italics. |
| First sentence under research design | Researchers (plural) has been used in the preceding paragraph but researcher (singular) has been used here. The same is repeated in the last paragraph and other pages of the manuscript. |
| Second paragraph under research design | Change “a) women **who have** mentally impaired” to “women **who are** mentally impaired”. |
| Line 3 of third paragraph under research design | “ … that were shown” make the sentence confusing. |
| Line 5 under third paragraph of research design | Capitalise the first letters of Ghana health service. That is “**Ghana Health Service**”. |
| Line 11 under third paragraph of research design | What sort of compensation was given to participants? Correct it if it is a mistake. |
| Last sentence of paragraph six of research design | Change the sentence from future tense to past tense. That is, “ … **will be** …” to “**was**”. |
| Line 2 of first paragraph under result section. | What does the figures 26 and 59 stand for? I presume they are age ranges. |
| Title of Table 1 | Add “of respondents”. That is Demographic characteristics **of respondents** |
| Last column of Table 1 | First income bracket should be 100-1000 and NOT 100-100.  Add the currency |
| Table 2 | The content is ambiguous. |
| First quotation from respondent (P 16) | Change “younger” to “young”. |
| Last sentence preceding quotation P 20  Under subheading, “ineffectiveness of antimalarial drug | Authors write “… prefer herbal remedies due to how ineffective the orthodox are.” This statement presumes orthodox medicine are ineffective. This statement is not backed by any scientific evidence. Authors should be mindful that their findings are based on perceptions of respondents. This should reflect in the sentence above. |
| Third line of quotation P 20 | “…when I take it …” is confusing. What does the “it” refers to? I suggest you replace “it” with “orthodox medicine” and use the pronoun later. |
| Last sentence of quotation P 20 | “ … because I felt dying…” should be change to “ … because I felt like dying…”  “ … I can’t get up …” should be change to “…. I couldn’t get up” |
| Fifth line of quotation P 14 | Change “**sit**” to “**sat**” |
| Eighth line of quotation P 6 | Change “… after I **take** towel off …” to “after I **took** …” |
| Second line under dosage for herbal medicine” section | Change “the dose for that remedy **was** determined by …” to “… that remedy **is** determined by …” |
| Second line of quotation P 13 | Change “… you separate the leaves **for** it” to “… you separate the leaves **from** it” |
| Quotation 20 under dosage for herbal medicine section | Change “what I know **off** is that…” to “what I know is that ... ” |
| Line 5 under “effectiveness of herbal treatment malaria treatment” section | Change “ … they would take herbal treatment **of** malaria over …” to “ they would take herbal treatment **for** malaria over …” |
| Line 7 of discussion section | Changed “ … **contracted** these results.” to “ … **contrasted** these results”. |
| Discussion | Authors could reflect on the influence of education and religion on the Ghanaian culture. |
| Conclusion | The conclusion should emanate from the research findings. What authors currently have written are problem statement and recommendations. |
