# Supplementary material for: Practices of herbal management of malaria among trading mothers in Shai Osudoku District, Accra
Source: PLoS One. 2022 Jul 18;17(7):e0271669. doi: 10.1371/journal.pone.0271669 (PMC9292099; doi:10.1371/journal.pone.0271669)
Supplement: Attachment — Submitted filename: RESPONSE TO REVIEWERS COMMENT.docx [file pone.0271669.s003.docx]

| REVIEWER | COMMENT | RESPONSE |
| --- | --- | --- |
| EDITOR | We note you have included a table to which you do not refer in the text of your manuscript. Please ensure that you refer to Tables 1 and 2  in your text; if accepted, production will need this reference to link the reader to the Table. | Table 1 and 2 have been included in the test as suggested |
| EDITOR | 3. In your Data Availability statement, you have not specified where the minimal data set underlying the results described in your manuscript can be found. PLOS defines a study's minimal data set as the underlying data used to reach the conclusions drawn in the manuscript and any additional data required to replicate the reported study findings in their entirety. All PLOS journals require that the minimal data set be made fully available. For more information about our data policy, please see http://journals.plos.org/plosone/s/data-availability. | All data are made available and attached as supplementary files |
| EDITOR | 4. Please include your full ethics statement in the ‘Methods’ section of your manuscript file. In your statement, please include the full name of the IRB or ethics committee who approved or waived your study, as well as whether or not you obtained informed written or verbal consent. If consent was waived for your study, please include this information in your statement as well. | Revision has been made as suggested |
| REVIEWER 1 | 1. Could there be a clear clarification and justification for why Trading mothers were used? And why the study area was selected among numerous markets in Ghana? | These trading mothers were selected from rural communities where malaria was prevalent and mothers who came to trade were from different communities in the district |
| REVIEWER 1 | 2. It is well established that there are four aspects of trustworthiness that qualitative researchers must establish: credibility, dependability, transferability, and confirmability. In the current study, the authors tried to explain how credibility was established ie explained as the accuracy and authenticity of a research finding. Credibility essentially asks the authors to clearly link their findings with reality in order to demonstrate the truth of the research study’s findings. This is not well clarified in the current version of the paper. Also, the other aspect of trustworthiness ie dependability, transferability and confirmability must clearly be explained in the paper. | This has been revised and shown in track changes |
| REVIEWER 1 | 3. The authors should be specific about which approach of content analysis did they use: conventional, directed, or summative? | Conventional approach was used and has been included as suggested |
| REVIEWER | 4. Result section, the second line … “The participants were between twenty-six (26) and fifty-nine (59)”?? Sentence is not complete. | This has been corrected |
| REVIEWER | 5. What does JSS, SSS and Form 4 level of education means? 6. Table 1..The Title should be made complete 7. Table 2…Table should be well formatted..lines are missing 8. Overall, the paper needs to be well formatted, for example, the section titles and Tables should follow the journal style. All the Tables should be cited in the text. | REVISION MADE TO THESE COMMENTS |
| Reviewer | Remove full stop after the title.  Change “the researchers, therefore aim … “ to “ The study aims at …”  The conclusion in the abstract is misplaced. Authors must summarize their findings  Complete the statistics on the number of deaths attributed to malaria. Is it per annum or monthly?  Scientific names of trees should be written in italics. | Full stop has been removed  This has been revised  The conclusion has been revised  This has been completed as suggested  Scientific names have been italicized |
|  | Researchers (plural) has been used in the preceding paragraph but researcher (singular) has been used here. The same is repeated in the last paragraph and other pages of the manuscript  Change “a) women **who have** mentally impaired” to “women **who are** mentally impaired”.  “ … that were shown” make the sentence confusing.  Capitalise the first letters of Ghana health service. That is “**Ghana Health Service**”.  What sort of compensation was given to participants? Correct it if it is a mistake.  Change the sentence from future tense to past tense. That is, “ … **will be** …” to “**was**”.  What does the figures 26 and 59 stand for? I presume they are age ranges.  Add “of respondents”. That is Demographic characteristics **of respondents**  First income bracket should be 100-1000 and NOT 100-100.  Add the currency  The content is ambiguous. | Revision has been made  This has been changed appropriately  It has been changed to “A tape recorder was used to capture the interviews during the recording  Corrected  This has been corrected  Corrected  Corrections made  Respondents have been added  Corrected  The content has been revised |
| REVIEWER | Change “younger” to “young”. | CORRECTED |
|  | Authors write “… prefer herbal remedies due to how ineffective the orthodox are.” This statement presumes orthodox medicine are ineffective. This statement is not backed by any scientific evidence. Authors should be mindful that their findings are based on perceptions of respondents. This should reflect in the sentence above. | It has been revised appropriately as suggested |
| REVIEWER | “…when I take it …” is confusing. What does the “it” refers to? I suggest you replace “it” with “orthodox medicine” and use the pronoun later. because I felt dying…” should be change to “ … because I felt like dying…” “ … I can’t get up …” should be change to “…. I couldn’t get up” Change “**sit**” to “**sat**” Change “… after I **take** towel off …” to “after I **took** …”; Change “the dose for that remedy **was** determined by …” to “… that remedy **is** determined by …” | Revised  Revision made  Revised  Revised |
| REVIEWER | Change “… you separate the leaves **for** it” to “… you separate the leaves **from** it”  Change “what I know **off** is that…” to “what I know is that ... ”  Change “ … they would take herbal treatment **of** malaria over …” to “ they would take herbal treatment **for** malaria over …”  Changed “ … **contracted** these results.” to “ … **contrasted** these results”. | This has been changed as suggested  Corrected  Changes have been made  This has been revised |
| REVIEWER | Authors could reflect on the influence of education and religion on the Ghanaian culture. | This has been included in the discussion |
| REVIEWER | The conclusion should emanate from the research findings. What authors currently have written are problem statement and recommendations. | The conclusion has been revised |
